# Extreme intracranial pressure elevation > 90 mmHg in an awake patient with primary CNS lymphoma—case report

**DOI:** 10.1007/s00701-020-04231-x

**Published:** 2020-01-22

**Authors:** David Cederberg, Niklas Marklund, Henrietta Nittby Redebrandt

**Affiliations:** Department of Clinical Sciences Lund, Neurosurgery, Skane University Hospital, Lund University, Lund, Sweden

**Keywords:** Intracranial pressure, Lymphoma, Intracranial hypertension

## Abstract

We describe a patient with primary CNS lymphomas, awake despite an extreme ICP elevation. A 48-year-old woman presented with headache since 1 month, and bilateral papillary edema was observed. Magnetic resonance imaging revealed diffuse infiltration around the petrous bone. Following external ventricular drainage (EVD) placement, ICP levels of > 90 mmHg were recorded while the patient was fully awake. Cytology revealed an aggressive primary CNS lymphoma. Cerebrospinal fluid (CSF) drainage at high opening pressure levels was required. We conclude that extreme ICP elevations, treatable by CSF drainage, can be observed without a reduced level of consciousness.

## Background and importance

Monitoring and treatment of elevated intracranial pressure (ICP) is an integral part of neurosurgical management of acute brain injury, including traumatic brain injury (TBI). Although normal ICP is commonly assumed to be ca 7–15 mmHg, recent data suggests that it may be as low as 0–5 mmHg [2]. When ICP increases, there is an increased risk of cerebral ischemia and herniation, and ultimately collapse of cerebral microcirculation [6]. Although survival has been observed in TBI patients with ICP ≥ 50 mmHg [14], increased ICP is consistently associated with poor outcome and high mortality [9, 12]. If neurosurgical and/or neurocritical care treatment can successfully lower an elevated ICP, prognosis is more favorable [3]. The threshold for initiating treatment of an elevated ICP is commonly set at 20–22 mmHg, although the duration and time of ICP increase (i.e., the “ICP dose”) [4, 13] may be critical.

Although high ICP is a common observation in acute brain injury, hydrocephalus, or bacterial meningitis [10], in certain disorders such as liver failure, extremely elevated ICP may be observed [8]. In cryptococcal meningitis, a lumbar cerebrospinal fluid (CSF) opening pressure of > 25 mmHg was negatively associated with long-term survival. Another condition associated with increased ICP is idiopathic intracranial hypertension (IIH), where a lumbar opening pressure of > 25 mmHg is required for diagnosis [11].

Primary CNS lymphoma (PCNSL) accounts for 1–5% of all brain tumors [5]. Symptoms of PCNSL may include headache, nausea, vomiting, papilledema, and lethargy [1] considered indirect signs of increased ICP and seen in approximately one-third of patients. However, ICP measurements have, to the best of our knowledge, not previously been reported.

We here describe a case of extremely high ICP of > 90 mmHg in a fully awake and orientated patient with PCNSL.

## Clinical presentation

**Fig. 1:**
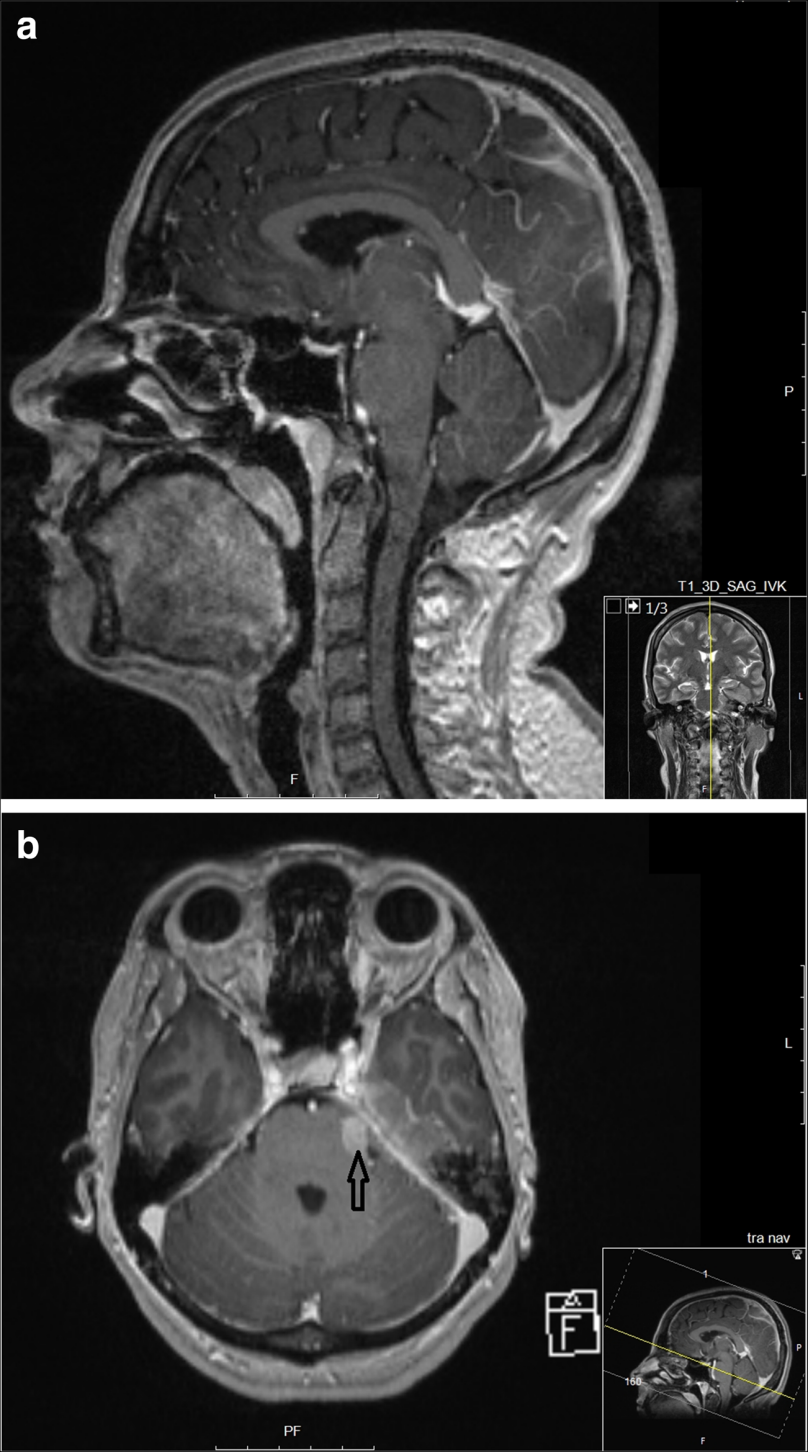
**a** Sagittal MRI scanning before initiation of oncological treatment. No sign of herniation was seen, and no sign of obstructive hydrocephalus. **b** Axial MRI scanning showing peri-pontine tumor infiltration (arrow)

A 48-year-old woman with no previous medical history was admitted to the emergency department due to headache for 1 month, and facial nerve palsy 4 days. Upon clinical examination, a peripheral facial nerve palsy, abducens nerve palsy, and decreased sensibility on the left side of the face mainly corresponding to the innervation area of nervus ophthalmicus were noted. The patient was fully conscious and orientated. A cerebellopontine angle pathology was suspected, and an emergent MRI was done revealing an unspecific infiltration around the apex pars petrosa (Fig. 1). Ophthalmoscopic examination revealed bilateral papillary edema and hemorrhages. A lumbar puncture in the lateral supine position was performed at the department of neurology, despite the presence of papillary edema, since the patient was fully awake, and an increased ICP was not suspected. An opening pressure of > 50 cm H20 (> 37 mmHg; higher values could not be measured) as well as elevated protein levels (0.83 g/L) and polymorphonuclear leukocyte count (617 × 10^ 6/L) were observed whereas the mononuclear leukocyte count was normal. Tuberculosis meningitis was suspected, and treatment was initiated.

**Fig. 2:**
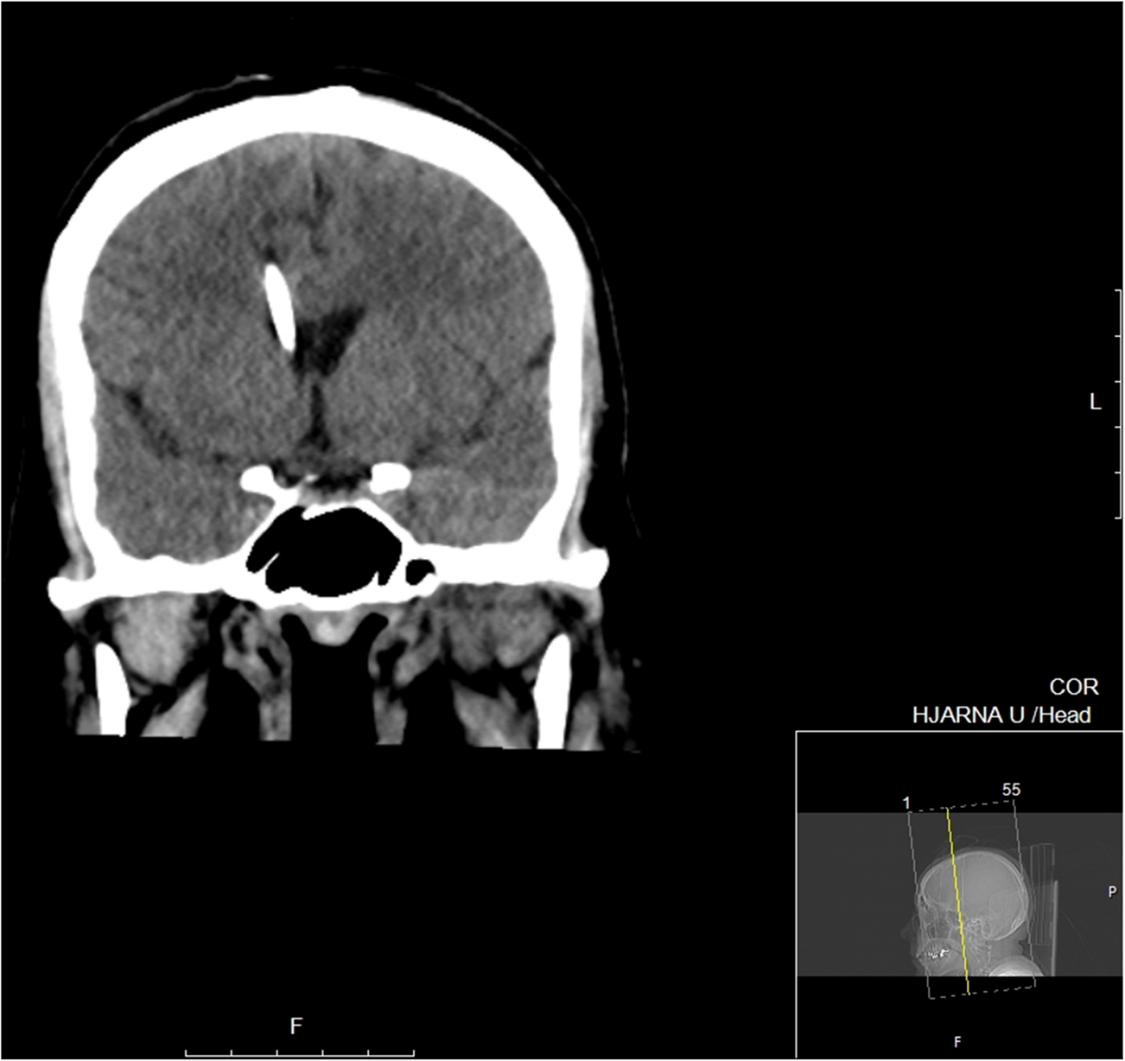
Coronal CT scanning with the EVD in place, with catheter tip at the level of foramen of Monroi (not seen in this projection). Extremely high ICP recordings at 52–60 mmHg was obtained within 30 min of this CT, and the blood pressure was 183/121, resulting in a CPP > 80. Later the same day, a total of 125 mL of CSF was drained through the EVD at an opening pressure of 60 mmHg

**Fig. 3:**
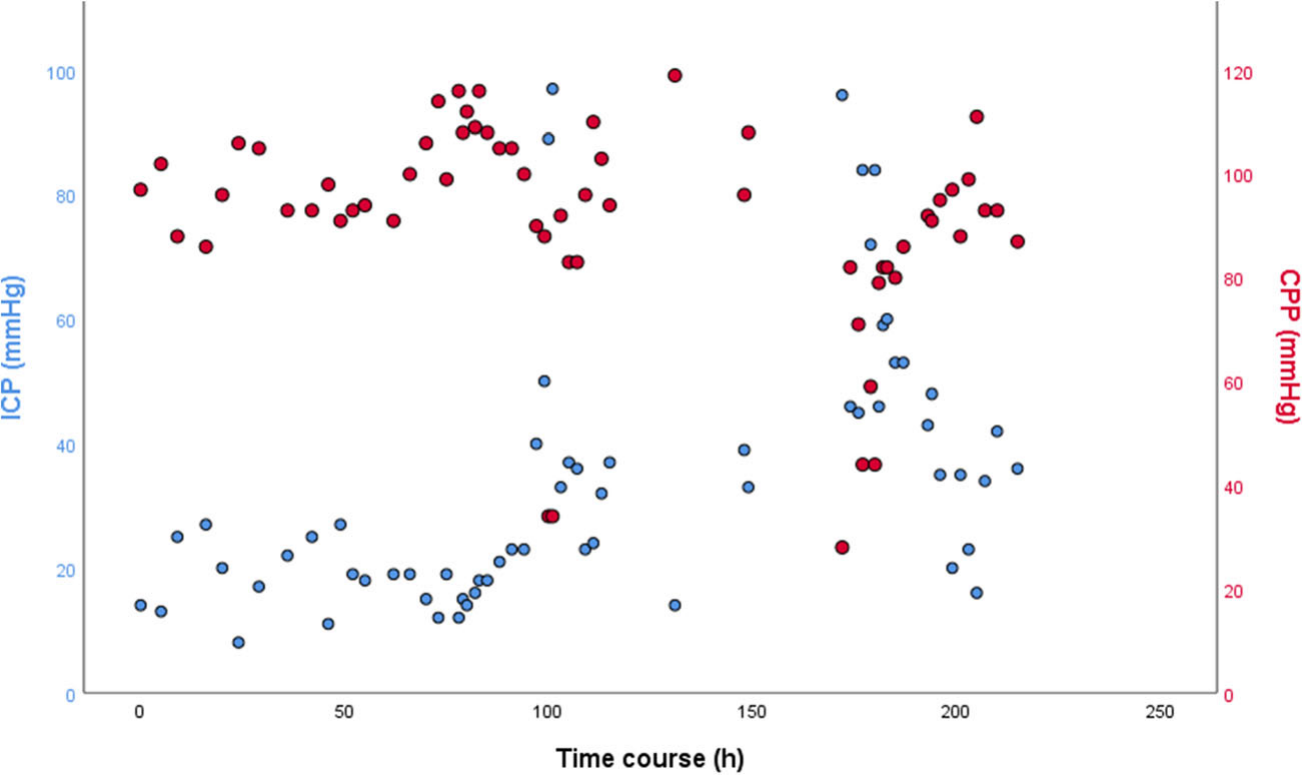
ICP and MAP co-registrations are shown. CPP is calibrated as MAP-ICP. ICP was measured from the EVD with reference level at the forehead (blue dots). Initially, the patient had an arterial line, which showed a MAP which was approximately 10 mmHg above the non-invasive blood pressure. However, due to problems to keep the arterial line working continuously, non-invasive blood pressure was recorded instead. Thus, CPP values registered here represent MAP calibrations made from non-invasive blood pressure (red dots). Initially, the EVD was open for CSF drainage, thus explaining the relatively low ICP values seen during the first days. As the patient was weaned from the CSF drainage, ICP increased as a result of increased EVD drainage resistance

Continuous CSF sampling was considered indicated to enable monitoring of the treatment response. Therefore, the patient was transferred to the department of neurosurgery, and an external ventricular drainage (EVD) was inserted (Fig. 2). The patient was fully awake and pupillary reactivity was preserved, and the EVD was initially planned to be used only for CSF sampling. However, when ICP was measured by closing the EVD on the first post-operative day (several hours after the EVD placement), calibrated to the level of the forehead, it read 96 mmHg. At this time, the patient was still fully awake and orientated (a Glasgow Coma Scale (GCS) score of 15). Lundberg type A waves/plateau waves were observed when evaluating the ICP wave form. Transcranial Doppler revealed a pulsatile index of 1.8, and the arterial blood pressure was 152/103 mmHg (MAP 119). Due to the high ICP and low cerebral perfusion pressure (CPP), the EVD was initially opened for CSF drainage at a level of 30 mmHg, and intravenous fluid was administered. Over the next days, the patient needed continuous CSF drainage since she developed a severe trigeminal neuralgia when the EVD was closed. Drainage of CSF was regulated by adjusting the height of the CSF sampling device above the patient’s head. On EVD closure, her ICP gradually increased to > 90 mmHg after 2 h; still, she was GCS 15 (Fig. 3).

CSF cytology revealed the presence of a highly malignant primary CNS B cell lymphoma. Intrathecal methotrexate was administered, and a chemotherapy treatment protocol was initiated. Initially, CSF drainage at an opening pressure of 30 mmHg was used, and the patient drained 188 mL during the first 24 h. On day 3, we used an opening pressure of 40 mmHg, draining 304 mL CSF, and on day 4, an opening pressure of 60 mmHg was used, draining 225 mL CSF. The drainage at 60 mmHg was maintained for 3 days, after which the EVD could be closed. Adequate pulsations were observed on the ICP curve. ICP was 95 mmHg initially, but then slowly decreased; however, it was only < 20 mmHg on four occasions. Co-registrations of CPP and ICP are presented in Fig. 3. The patient had less facial pain, and the EVD could be converted to a Rickham reservoir, which was used for further intrathecal chemotherapy (methotrexate) treatment although not ICP measurements at the department of oncology for further treatment. No ventriculoperitoneal shunting was needed, and the patient had no neurologic deficits and experienced complete remission of her PCNSL. However, she suffered from an early recurrence of the disease 5 months later, with extracerebral metastases. Further chemotherapy led to neutropenia and sepsis, and the patient died shortly thereafter.

## Discussion and conclusions

The present case highlights that (i) ICP can be extremely elevated in certain disorders, even in a fully awake patient, (ii) PCNSL can lead to marked ICP elevations, and (iii) CSF drainage via an open EVD can be a useful treatment strategy when an extreme ICP elevation is encountered. Here, no cerebral ischemia or radiological signs of increased intracerebral pressure were observed on neuroimaging. We carefully searched for sources of erroneous measurement. Furthermore, the EVD system was routinely recalibrated on a daily basis. The patient drained CSF at high resistance values, controlled by manually adjusting the level of the CSF sampling device, strongly arguing that the measured ICP values were correct. ICP measurements were done by temporarily closing the EVD. Since the patient needed CSF drainage for ICP control, there was no continuous measurement of ICP. Therefore, the presence of Lundberg A waves could not be determined, although were not noted. Plausibly, the patient had a sufficiently high CPP and cerebral blood flow, stressing the importance of maintaining a high MAP in the event of critically elevated ICP. Previous reports on traumatic brain patients with severely elevated ICP have provided some support that outcome can be favorable even at an ICP > 40 mmHg when maintaining a CPP > 60 mmHg [3]. We speculate that the high ICP observed here was tolerated since it (a) plausibly developed over a long time, (b) was found without any focal lesion, and (c) the CPP was maintained over critical levels.

In CNS lymphoma, similar ICP elevations have not previously been reported to our knowledge. In other forms of cancer, high ICP has been observed, such as in a case of a fully alert young woman presenting with headache, vomiting, and papilledema secondary to a gastric signet-ring cell carcinoma. Here, ICP was > 29 mmHg, and meningeal carcinomatosis was suspected [7].

Our report emphasizes the need for ophthalmological evaluation when a patient presents with clinical signs of ICP elevation, even in the absence of obvious radiological findings. The cause of the extremely high ICP in the present case is unclear, without signs of cerebral edema, although infiltration of lymphoma cells obstructing CSF resorption and leading to communicating hydrocephalus is a plausible explanation. Drainage of CSF at high resistance under careful monitoring of the neurological state appears acceptable when facing extreme ICP elevations without midline shift or focal mass lesions.

## References

[1] Algazi AP, Kadoch C, Rubenstein JL (2009). Biology and treatment of primary central nervous system lymphoma. Neurotherapeutics.

[2] Andresen M, Juhler M (2014). Intracranial pressure following complete removal of a small demarcated brain tumor: a model for normal intracranial pressure in humans. J Neurosurg.

[3] Farahvar A, Gerber LM, Chiu YL, Härtl R, Froelich M, Carney N, Ghajar J (2011). Response to intracranial hypertension treatment as a predictor of death in patients with severe traumatic brain injury. J Neurosurg.

[4] Flechet M, Meyfroidt G, Piper I, Citerio G, Chambers I, Jones PA, et al. (2018) Visualizing cerebrovascular autoregulation insults and their association with outcome in adult and paediatric traumatic brain injury. Acta Neurochir Suppl.126:291-295 10.1007/978-3-319-65798-1_57 29492577

[5] Haldorsen IS, Espeland A, Larsson EM (2011). Central nervous system lymphoma: characteristic findings on traditional and advanced imaging. Am J Neuroradiol.

[6] Harary M, Dolmans RGF, Gormley WB (2018) Intracranial pressure monitoring-review and avenues for development. Sensors (Basel) 18(2) 10.3390/s18020465 PMC5855101 29401746

[7] Jiali P, Linglia X, Xinzhen Y, Baorong Z (2016). Intracranial hypertension as the primary symptom of gastric signet-ring cell carcinoma. Medicine.

[8] Maloney PR, Mallory GW, Atkinson JL, Wijdicks EF, Rabinstein AA, Van Gompel JJ (2016). Intracranial pressure monitoring in acute liver failure: institutional case series. Neurocrit Care.

[9] Nourallah B, Zeiler FA, Calviello L, Smielewski P, Czosnyka M, Menon DK (2018). Critical thresholds for intracranial pressure vary over time in non-craniectomised traumatic brain injury patients. Acta Neurochir.

[10] Tariq A, Aguilar-Salinas P, Hanel RA, Naval N, Chmayssani M (2017). The role of ICP monitoring in meningitis. Neurosurg Focus.

[11] Thurtell MJ, Bruce BB, Newman NJ, Biousse V (2010). An update on idiopathic intracranial hypertension. Rev Neurol Dis.

[12] Treggiari MM, Schutz N, Yanez ND, Romand JA (2007). Role of intracranial pressure values and patterns in predicting outcome in traumatic brain injury: a systematic review. Neurocrit Care.

[13] Vik A, Nag T, Fredriksli OA, Skandsen T, Moen KG, Schirmer-Mikalsen K, Manley GT (2008). Relationship of “dose” of intracranial hypertension to outcome in severe traumatic brain injury. J Neurosurg.

[14] Young JS, Blow O, Turrentine F, Claridge JA, Schulman A (2003). Is there an upper limit of intracranial pressure in patients with severe head injury if cerebral perfusion pressure is maintained?. Neurosurg Focus.

